# TP53 isoform junction reads based analysis in malignant and normal contexts

**DOI:** 10.1038/s41598-021-96700-1

**Published:** 2021-08-26

**Authors:** Suleyman Vural, Lun-Ching Chang, Laura M. Yee, Dmitriy Sonkin

**Affiliations:** 1 National Cancer Institute, Division of Cancer Treatment and Diagnosis, Biometric Research Program, Rockville, MD 20850 USA; 2 Department of Mathematical Sciences, Florida Atlantic University, Boca Raton, FL 33431 USA; 3 Present Address: Memorial Sloan Kettering Cancer Center, New York, NY USA

**Keywords:** Cancer, Cancer genomics, Genome informatics

## Abstract

TP53 is one of the most frequently altered genes in cancer; it can be inactivated by a number of different mechanisms. NM_000546.6 (ENST00000269305.9) is by far the predominant TP53 isoform, however a few other alternative isoforms have been described to be expressed at much lower levels. To better understand patterns of TP53 alternative isoforms expression in cancer and normal samples we performed exon-exon junction reads based analysis of TP53 isoforms using RNA-seq data from The Cancer Genome Atlas (TCGA), Cancer Cell Line Encyclopedia (CCLE), and Genotype-Tissue Expression (GTEx) project. TP53 C-terminal alternative isoforms have abolished or severely decreased tumor suppressor activity, and therefore, an increase in fraction of TP53 C-terminal alternative isoforms may be expected in tumors with wild type TP53. Despite our expectation that there would be increase of fraction of TP53 C-terminal alternative isoforms, we observed no substantial increase in fraction of TP53 C-terminal alternative isoforms in TCGA tumors and CCLE cancer cell lines with wild type TP53, likely indicating that TP53 C-terminal alternative isoforms expression cannot be reliably selected for during tumor progression.

## Introduction

Tumor suppressor gene TP53 plays an important role in tumor biology and has been extensively studied since its discovery about 40 years ago^1^. TP53 can be inactivated by a variety of different mechanisms such as missense loss of function mutations, frame shift and nonsense mutations, splice site mutations, deletions, rearrangements, and loss of expression^2–4^. To fulfill its proper biological function four TP53 polypeptides must form a tetramer which functions as a transcription factor^5,6^. Therefore, even if one out of four polypeptides have an inactivating mutation it may lead to a dominant negative phenotype of variable degree due to effects on the tetramer^2,7^.

**Figure 1:**
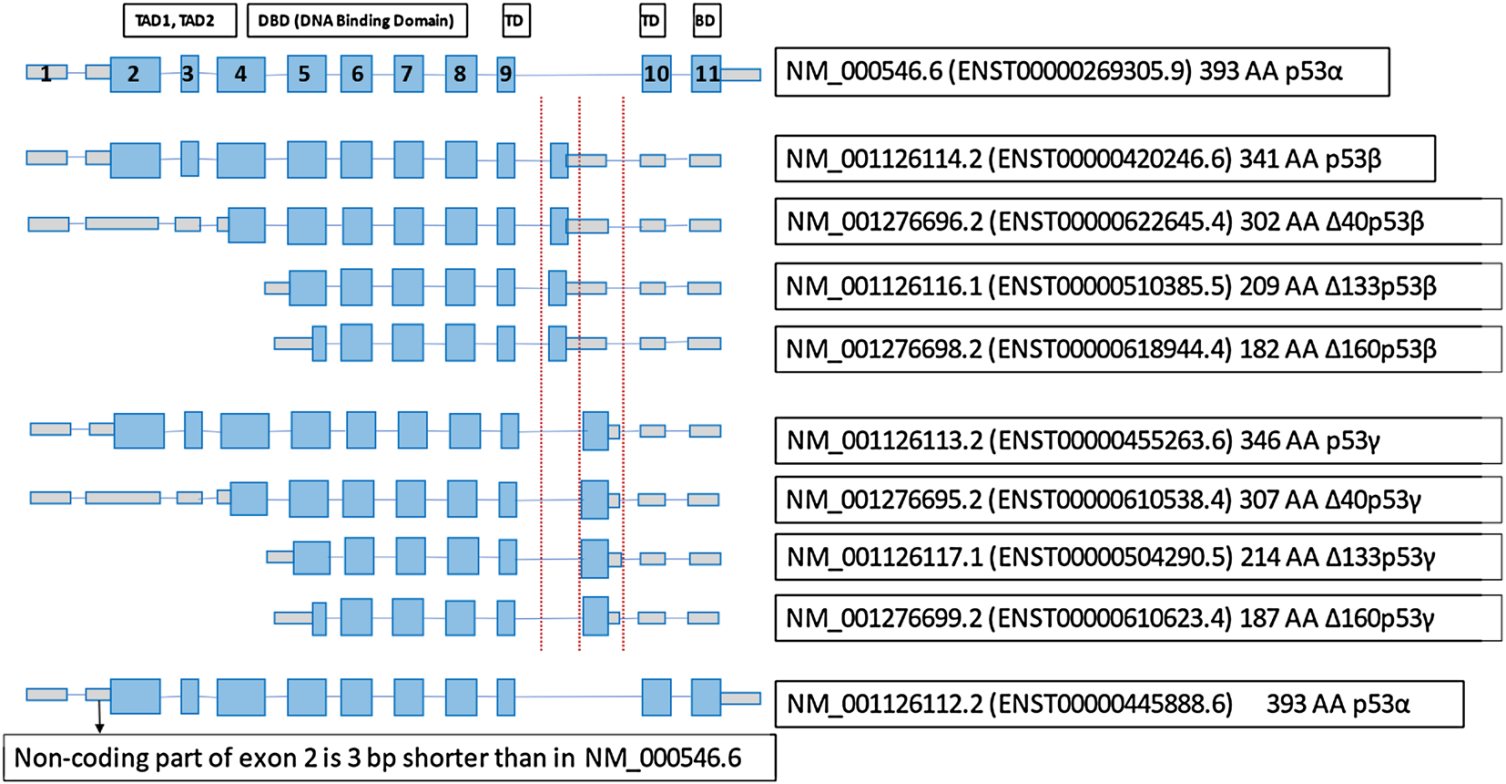
TP53 isoforms with exon-exon junctions distinct from main NM_000546.6 isoform. TAD—Trans Activation Domain, TD—Tetramerization Domain, BD—Basic Domain.

NM_000546.6 (ENST00000269305.9), also known as p53α, is by far the predominant TP53 isoform, however a few other alternative isoforms have been described to be expressed at much lower levels^8–10^. To better understand patterns of TP53 alternative isoforms expression in cancer and normal samples we performed exon-exon junction reads based analysis of TP53 isoforms using RNA-seq data from The Cancer Genome Atlas (TCGA), Cancer Cell Line Encyclopedia (CCLE), and Genotype-Tissue Expression (GTEx) projects. Exon-exon junction reads based analysis allows us to look for unambiguous evidence of junctions between two exons using short reads RNA sequencing data. In order to differentiate between predominant TP53 isoform NM_000546.6 and other alternative isoforms, this analysis was focused on exon-exon junctions unique to alternative isoforms and not present in predominant TP53 isoform NM_000546.6. Figure 1 illustrates such exon junctions. Unfortunately, TP53 N-terminal alternative isoforms do not have unique exon-exon junctions and therefore analysis is limited to TP53 C-terminal alternative isoforms.

Following alternative TP53 isoforms are depicted in Fig. 1: NM_001126114.2 (ENST00000420246.6) also known as p53β, NM_001276696.2 (ENST00000622645.4) also known as ∆40p53β, NM_001126116.1 (ENST00000510385.5) also known as ∆133p53β, NM_001276698.2 (ENST00000618944.4) also known as ∆160p53β, NM_001126113.2 (ENST00000455263.6) also known as p53γ, NM_001276695.2 (ENST00000610538.4) also known as ∆40p53γ, NM_001126117.1 (ENST00000504290.5) also known as ∆133p53γ, NM_001276699.2 (ENST00000610623.4) also known as ∆160p53γ, and NM_001126112.2 (ENST00000445888.6). In GTEx, TCGA and CCLE RNA-seq data exon-exon junctions supporting presence of p53γ/∆40p53γ/∆133p53γ/∆160p53γ isoforms are very rare, therefore, the following analysis using exon-exon junction reads is focused on isoforms p53β/∆40p53β/∆133p53β/∆160p53β and NM_001126112.2. Isoforms p53β/∆40p53β/∆133p53β/∆160p53β all have the same C-terminal alternation with specific exon-exon junction, which is used for exonic fraction calculations. For brevity, we refer to these isoforms as C-terminal isoforms. TP53 C-terminal isoforms do not have functional tetramerization domain, therefore four TP53 polypeptides cannot form a tetramer which is essential for TP53 function, these isoforms are considered to be non-functional or at a minimum with severely diminished functionality^10^. NM_001126112.2 uses an alternate splice site in the 5' UTR resulting in loss of 3 bp in the non-coding part of exon 2 in comparison to NM_000546.6, however it encodes exactly the same protein sequence as NM_000546.6.

We used exon-exon junctions unique to C-terminal alternative isoforms and not present in predominant TP53 isoform NM_000546.6 as outlined in Fig. 1 (exons between 3 vertical dotted red lines) to perform exon-exon junction reads based analysis of TP53 C-terminal isoforms using RNA-seq data from TCGA, CCLE, and GTEx projects.

## Materials and methods

RNA-seq data (101 base pairs read length) from 834 CCLE cancer cell lines, corresponding TP53 mutation calls based on DNA and/or RNA sequencing, and TP53 CN-ratio based on AFFY SNP 6.0 arrays have been used in analysis^11^. (CN-ratio derived from AFFY SNP 6.0 arrays is the ratio of signal intensity in a tumor sample versus normal reference samples normalized to total DNA quantity; thus, a CN-ratio of 1 corresponds to a diploid locus.) TCGA RNA-seq data (48 to 76 base pairs read length) from 8795 samples with corresponding TP53 mutation calls and arrays based TP53 CN-ratio has been used in this analysis^12,13^. GTEx RNA-seq data (76 base pairs read length) from 9512 normal tissue samples from 549 donors has been used in analysis^14^. RNA-seq data (BAM files) from GTEx, TCGA, CCLE were used as provided by corresponding sources.

**Figure 2:**
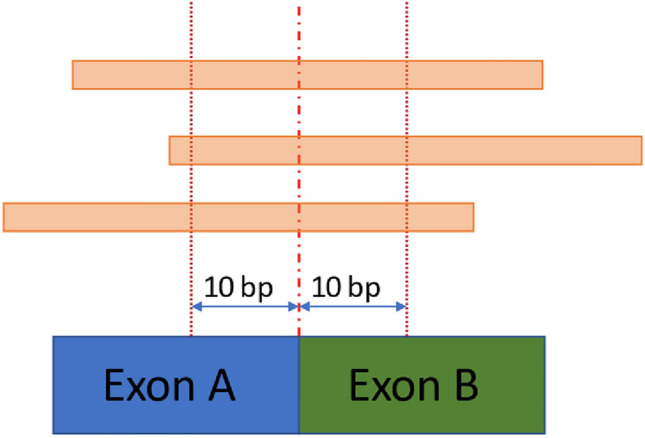
Exon-exon junctions reads.

Reads are considered as exon-exon junction reads if the aligned read has no mismatches (no more than half of read could be soft clipped), has a breakpoint exactly matching expected exon-exon boundary, and the read has at least ten nucleotides on both sides of breakpoint as illustrated on Fig. 2.

Exonic fraction of junctions distinct from TP53 main isoform is calculated as (number of distinct junctions reads between relevant exons) / (number of TP53 main isoform junctions reads between relevant exons + number of distinct junctions reads between relevant exons) for each sample selected for analysis. In isoform p53β (NM_001126114.2) junctions between exon 9 and exon 10, exon 10 and exon 11 are distinct from TP53 main isoform NM_000546.6 junction between exon 9 and exon 10, for better comparison number of junction reads between exon 9 and exon 10, exon 10 and exon 11 in isoform NM_001126114.2 is averaged for exonic fraction calculations. This particular exon-exon junction is also present in ∆40p53β/∆133p53β/∆160p53β isoforms and is used to calculate exonic fraction for C-terminal isoforms.

Statistical analysis is exploratory in nature, and is meant to describe the datasets at hand. Mean and median isoform fractions are calculated, as appropriate. P-values are assessed at an adjusted significance level, where the adjustment to the significance level is made to account for multiple comparisons using a Bonferroni correction. The number of t-tests performed in the primary analysis of the TCGA data in Tables 2 and 3 is 79 and therefore the Bonferroni adjusted significance level for Tables 2 and 3 is ~ 6.33E–4. The number of *t *tests performed in the secondary analysis of the TCGA data in Supplemental Table 4 and Supplemental Table 5 is 76 and therefore the Bonferroni adjusted significance level for Supplemental Table 4 and Supplemental Table 5 is ~ 6.58E–4. Number of *t *tests performed in the analysis of CCLE data in Supplemental Table 7 is 10 and therefore the Bonferroni adjusted significance level for Supplemental Table 7 is 0.005. *P* values are considered to be statistically significant only if they meet these adjusted significance levels.

## Results

As a first step in our analysis we used GTEx RNA-seq data in order to measure fraction of C-terminal and NM_001126112.2 alternative isoforms mRNA expression in normal tissues using exon-exon junctions reads based approach. Details on calculating exonic fraction of exon-exon junctions is described in materials and methods. Table 1 lists exonic fraction average and median for alternative C-terminal isoforms across GTEx tissue types. (Supplemental Table 1 provides exonic fractions for C-terminal and NM_001126112.2 alternative isoforms for each GTEx sample used in analysis.) As can be seen from Table 1, the highest median percentage for C-terminal alternative isoforms expression is ~ 4% in skin and spleen. The average percentage for C-terminal alternative isoforms expression across all GTEx tissue types is ~ 2%. NM_001126112.2 expression is a few times lower than of C-terminal alternative isoforms, with highest expression also observed in skin. Similar patterns for C-terminal and NM_001126112.2 alternative isoforms expression are observed in gtexportal.org.

**Table 1:** Exonic fraction average and median percentage for C-terminal alternative isoforms across GTEx tissue types.

GTEx tissue name	Median C-terminal alternative isoforms fraction	Mean C-terminal alternative isoforms fraction	Sample count
Spleen	0.042	0.0539	118
Skin–Not Sun Exposed (Suprapubic)	0.041	0.0579	271
Skin–Sun Exposed (Lower leg)	0.041	0.0509	397
Small Intestine–Terminal Ileum	0.035	0.0699	104
Whole Blood	0.034	0.0651	449
Fallopian Tube	0.031	0.0276	7
Breast–Mammary Tissue	0.029	0.0373	218
Bladder	0.029	0.0444	10
Cervix–Ectocervix	0.028	0.0367	6
Lung	0.024	0.0453	372
Minor Salivary Gland	0.023	0.0340	70
Adipose–Subcutaneous	0.022	0.0345	385
Cells–EBV-transformed lymphocytes	0.014	0.0154	138
Colon–Transverse	0.013	0.0214	202
Stomach	0.011	0.0287	203
Esophagus–Mucosa	0.010	0.0162	330
Thyroid	0.000	0.0260	359
Testis	0.000	0.0205	202
Ovary	0.000	0.0123	108
Vagina	0.000	0.0174	97
Heart–Atrial Appendage	0.000	0.0185	216
Brain–Caudate (basal ganglia)	0.000	0.0027	133
Esophagus–Muscularis	0.000	0.0184	282
Brain–Putamen (basal ganglia)	0.000	0.0002	101
Brain–Anterior cingulate cortex (BA24)	0.000	0.0058	95
Adrenal Gland	0.000	0.0132	159
Brain–Cerebellum	0.000	0.0044	128
Cervix–Endocervix	0.000	0.0120	5
Brain–Cerebellar Hemisphere	0.000	0.0071	93
Artery–Coronary	0.000	0.0226	140
Liver	0.000	0.0174	135
Esophagus–Gastroesophageal Junction	0.000	0.0136	176
Brain–Hippocampus	0.000	0.0011	99
Brain–Hypothalamus	0.000	0.0049	97
Prostate	0.000	0.0186	118
Brain–Amygdala	0.000	0.0019	78
Pancreas	0.000	0.0271	196
Heart–Left Ventricle	0.000	0.0163	266
Brain–Cortex	0.000	0.0027	130
Muscle–Skeletal	0.000	0.0180	468
Brain–Spinal cord (cervical c-1)	0.000	0.0085	70
Uterus	0.000	0.0110	90
Brain–Nucleus accumbens (basal ganglia)	0.000	0.0037	121
Pituitary	0.000	0.0061	124
Artery–Aorta	0.000	0.0224	246
Kidney–Cortex	0.000	0.0169	36
Brain–Frontal Cortex (BA9)	0.000	0.0055	116
Adipose–Visceral (Omentum)	0.000	0.0286	234
Cells–Transformed fibroblasts	0.000	0.0059	305
Artery–Tibial	0.000	0.0212	362
Colon–Sigmoid	0.000	0.0164	173
Nerve–Tibial	0.000	0.0178	334
Brain–Substantia nigra	0.000	0.0063	69

TCGA has subset of samples in which in addition to tumor sample there is matching adjacent normal tissue sample (Supplemental Table 2). Such paired samples present a valuable opportunity to compare C-terminal and NM_001126112.2 alternative isoforms expression in wild-type (WT) TP53 tumors and matching adjacent normal tissue. We used data from Supplemental Table 2 to select paired samples with tumor samples without TP53 mutations, we considered such tumors to be TP53 WT if they also exhibited a log2(CN-ratio) > − 0.9 and TP53 mRNA RNA-Seq V2 RSEM normalized > 300^12^. C-terminal alternative isoforms fraction average is ~ 0.62% in paired TP53 WT tumors and ~ 0.66% in paired adjacent normal tissues; there is no statistically significant increase in C-terminal isoforms presence in TP53 WT tumors in comparison to paired adjacent normal tissues, paired *t *test *p* value 0.689. NM_001126112.2 alternative isoform fraction average is ~ 1.29% in paired TP53 WT tumors and ~ 1.27% in paired adjacent normal tissues; there is no statistically significant difference in NM_001126112.2 isoform presence between paired TP53 WT tumors and adjacent normal tissues, paired *t *test *p *value 0.94.

We also used data from Supplemental Table 2 to compare paired TP53 tumors with frame shift, nonsense, splice site mutations and adjacent normal tissues. C-terminal alternative isoforms fraction average is ~ 2.4% in paired tumors TP53 with frame shift, nonsense, splice site mutations and ~ 0.9% in paired adjacent normal tissues, which corresponds to a statistically significant difference in C-terminal isoform presence between paired tumors with TP53 frame shift, nonsense, splice site mutations and adjacent normal tissues, paired *t* test *p* value 0.0053. This difference is likely driven by some of frame shift, nonsense, splice site mutations causing aberrant C-terminal splicing. NM_001126112.2 alternative isoform fraction average is ~ 0.85% in paired tumors with TP53 frame shift, nonsense, splice site mutations and ~ 1.66% in paired adjacent normal tissues; there is no statistically significant difference in NM_001126112.2 isoform presence between paired tumors with TP53 frame shift, nonsense, splice sites mutations and adjacent normal tissues, paired *t *test *p *value 0.056. This is likely due to vast majority of frame shift, nonsense, splice site mutations located after NM_001126112.2 isoform specific exon-exon junction in 5’ UTR.

**Table 2:** Comparison of exonic fractions for C-terminal alternative isoforms across TCGA tumor types between TP53 WT tumors and TP53 tumors with missense mutations.

Tumor type	TP53 WT tumors C-terminal alternative isoforms fraction mean	TP53 tumors with missense mutations C-terminal alternative isoforms fraction mean	*T* test *p* value	TP53 WT tumors sample count	TP53 tumors with missense mutations sample count
LUSC	0.014	0.008	0.191	38	246
HNSC	0.013	0.007	0.069	119	173
LIHC	0.012	0.008	0.664	209	61
STAD	0.010	0.008	0.165	168	102
ESCA	0.009	0.008	0.767	16	79
BLCA	0.009	0.007	0.422	184	123
KIRC	0.007	0.012	0.500	314	5
PAAD	0.007	0.004	0.257	58	61
LUAD	0.007	0.006	0.880	221	154
SKCM	0.007	0.007	0.939	283	35
CESC	0.006	0.009	0.503	247	12
COAD	0.005	0.004	0.251	156	153
BRCA	0.005	0.004	0.206	600	199
MESO	0.005	0.007	0.469	66	10
PRAD	0.004	0.004	0.747	407	40
UCEC	0.004	0.003	0.778	298	132
GBM	0.004	0.004	0.953	89	36
SARC	0.003	0.002	0.220	93	46
KICH	0.003	0.004	0.915	37	11
READ	0.003	0.004	0.700	26	69
ACC	0.003	0.011	0.085	56	5
LGG	0.002	0.003	0.027	248	179

TCGA samples are from 33 different tumor types, keeping in mind differences in C-terminal and NM_001126112.2 alternative isoforms expression across different tissue types, we performed comparisons between TP53 WT tumors (no TP53 mutations, log2(CN-ratio) > − 0.9 and TP53 mRNA RNA-Seq V2 RSEM normalized > 300) and tumors with TP53 missense mutations in each tumor type with at least 5 samples in each group. Supplemental Table 3 provides exonic fractions for C-terminal and NM_001126112.2 alternative isoforms for each TCGA sample used in this analysis. Table 2 provides comparison results for C-terminal alternative isoforms for 22 TCGA tumor types with sufficient number of samples. As can be seen from Table 2 across all 22 tumor types, there is no statistically significant increase in C-terminal alternative isoforms in TP53 WT tumors in comparison to tumors with TP53 missense mutations. As can be seen from Supplemental Table 4 across all 22 tumor types there is no statistically significant difference in NM_001126112.2 alternative isoform in TP53 WT tumors in comparison to tumors with TP53 missense mutations.

We also performed comparisons between TP53 WT tumors (no TP53 mutations, log2(CN-ratio) > − 0.9 and TP53 mRNA RNA-Seq V2 RSEM normalized > 300) and tumors with TP53 frame shift, nonsense, splice site mutations across 19 TCGA tumor types with sufficient number of samples. As can be seen from Table 3, in many tumor types, there is a statistically significant difference in C-terminal isoforms presence between tumors with TP53 frame shift, nonsense, splice site mutations and TP53 WT tumors. This difference is likely driven by some of frame shift, nonsense, splice site mutations causing aberrant C-terminal splicing.

**Table 3:** Comparison of exonic fractions for C-terminal alternative isoforms across TCGA tumor types between TP53 WT tumors and TP53 tumors with frame shift, nonsense, splice sites mutations.

Tumor type	TP53 WT tumors C-terminal alternative isoforms fraction mean	TP53 tumors with nonsense/splice site/frame shift mutations C-terminal alternative isoforms fraction mean	t-test p-value TP53 WT tumors vs tumors with nonsense/splice site/frame shift mutations	TP53 WT tumors sample count	TP53 tumors with nonsense/splice site/frame shift mutations sample count
LUSC	0.014	0.028/0.054/0.029	2.07E–01/1.41E–02/1.51E–01	38	48/44/57
HNSC	0.013	0.032/0.046/0.026	1.15E–02/6.22E–04/9.07E–02	119	62/31/60
LIHC	0.012	0.024/0.04/0.03	5.65E–01/2.94E–01/3.26E–01	209	12/7/16
STAD	0.010	0.03/0.046/0.034	1.19E–05/4.52E–07/5.58E–07	168	24/17/31
ESCA	0.009	0.04/0.058/0.053	1.98E–03/1.31E–02/5.44E–03	16	26/12/13
BLCA	0.009	0.056/0.209/0.032	1.79E–04/7.09E–17/9.67E–05	184	41/7/19
PAAD	0.007	0.004/0.012/0.003	2.73E–01/4.43E–01/2.23E–01	58	17/4/18
LUAD	0.007	0.025/0.043/0.016	4.78E–05/2.26E–13/1.12E–03	221	49/28/28
SKCM	0.007	0.035/0.014/0.004	1.48E–10/1.87E–01/7.41E–01	283	20/7/3
CESC	0.006	0.004/NA/0	7.15E–01/NA/NA	247	6/0/1
COAD	0.005	0.02/0.057/0.01	1.90E–06/4.66E–16/7.59E–02	156	27/10/21
BRCA	0.005	0.019/0.031/0.018	1.67E–14/1.73E–11/5.61E–15	600	47/24/66
PRAD	0.004	0.02/0.011/0.023	1.34E–02/5.14E–02/1.98E-07	407	2/7/9
UCEC	0.004	0.029/0.01/0.017	1.94E–05/3.14E–01/4.51E–03	298	20/7/20
GBM	0.004	0.014/0.008/0.025	4.88E–04/1.72E–01/1.49E–07	89	5/3/4
SARC	0.003	0.013/0.001/0	2.07E–02/4.08E–01/1.89E–01	93	10/10/12
KICH	0.003	0/0.013/0	3.88E–01/1.03E–01/NA	37	5/4/1
READ	0.003	0.016/0.006/0.045	5.58E–04/3.21E–01/4.88E–03	26	14/7/9
LGG	0.002	0.005/0.006/0.007	2.63E–02/4.62E–03/1.36E–03	248	18/14/24

As can be seen from Supplemental Table 5 in all, but one of the 18 tumor types there is no statistically significant difference (after correction for multiple hypothesis testing using Bonferroni method, as described in the Materials and Methods section) in NM_001126112.2 alternative isoform in TP53 WT tumors in comparison to tumors with TP53 frame shift, nonsense, splice site mutations. (The statistically significant difference in one tumor type is potentially an artifact due to small sample size.) This is likely because the vast majority of frame shift, nonsense, splice site mutations are located after the NM_001126112.2 isoform specific exon-exon junction in 5’ UTR.

CCLE is a well characterized collection of cancer cell lines with comprehensive genomic data which allows us to investigate patterns of TP53 C-terminal and NM_001126112.2 alternative isoforms expression. Supplemental Table 6 provides exonic fractions for C-terminal and NM_001126112.2 alternative isoforms, TP53 status and other relevant detailed data for each cell line. We performed comparisons between TP53 WT CCLE cell lines and CCLE cell lines with TP53 missense mutations with RNA-seq data. C-terminal alternative isoforms fraction average is ~ 3.63% in TP53 WT cell lines and ~ 3.64% in cell lines with TP53 missense mutations; there is no statistically significant increase in C-terminal isoform presence in TP53 WT cell lines in comparison to cell lines with TP53 missense mutations, *t* test *p *value 0.988 (2 tails, unequal variance). NM_001126112.2 alternative isoform fraction average is ~ 0.82% in TP53 WT cell lines and ~ 0.88% in cell lines with TP53 missense mutations; there is no statistically significant difference in NM_001126112.2 isoform presence in TP53 WT cell lines in comparison to cell lines with TP53 missense mutations, *t* test *p* value 0.287 (2 tails, unequal variance).

We also performed comparisons between TP53 WT cell lines and cell lines with TP53 frame shift, nonsense, splice site mutations. C-terminal alternative isoforms fraction average is ~ 23.95% in cell lines with TP53 frame shift, nonsense, splice site mutations; there is a statistically significant difference in C-terminal isoforms presence between cell lines with TP53 frame shift, nonsense, splice site mutations and TP53 WT cell lines, *t* test *p* value 6.62E–27 (2 tails, unequal variance). NM_001126112.2 alternative isoform fraction average is ~ 1.2% in cell lines with TP53 frame shift, nonsense, splice site mutations; there is no statistically significant difference in NM_001126112.2 isoform presence in TP53 WT cell lines in comparison to cell lines with TP53 frame shift, nonsense, splice site mutations, *t* test *p* value 0.12 (2 tails, unequal variance). Supplemental Table 7 summarizes patterns of TP53 C-terminal and NM_001126112.2 alternative isoforms expression.

## Conclusions

TP53 C-terminal alternative isoforms have abolished or severely decreased tumor suppressor activity, and therefore an increase in fraction of TP53 C-terminal alternative isoforms may be expected in tumors with wild type TP53. However, as we described in the results section, we observed no substantial increase in fraction of TP53 C-terminal alternative isoforms in TCGA tumors and CCLE cancer cell lines with wild type TP53, likely indicating that TP53 C-terminal alternative isoforms expression cannot be reliably selected for during tumor progression. Small, but noticeable C-terminal alternative isoforms expression differences across GTEx tissue types coupled with our observation that TP53 C-terminal alternative isoforms expression cannot be reliably selected for during tumor progression hints at the possibility that function of TP53 C-terminal alternative isoforms may lay in fine tuning TP53 activity. It is also interesting to note that presence of TP53 C-terminal alternative isoforms specific exon-exon junctions in TCGA tumors and in CCLE cancer cell lines is driven in part by tumors with frame shift, nonsense, splice site mutations causing in some cases aberrant C-terminal splicing.

## Supplementary Information

Supplementary Tables.

Supplementary Legends.
