# Supplementary material for: TP53 isoform junction reads based analysis in malignant and normal contexts
Source: Sci Rep. 2021 Aug 26;11:17275. doi: 10.1038/s41598-021-96700-1 (PMC8390641; doi:10.1038/s41598-021-96700-1)
Supplement: Supplementary file 2 — Supplementary Legends. [file 41598_2021_96700_MOESM2_ESM.docx]

**TP53 isoform junction reads based analysis in malignant and normal contexts**

**Supplementary table captions**

**Authors:** Suleyman Vural^1+^, Lun-Ching Chang^2^, Laura M. Yee^1^, Dmitriy Sonkin^1*^

^1^ National Cancer Institute, Division of Cancer Treatment and Diagnosis, Biometric Research Program, Rockville, MD 20850, USA; ^2^ Department of Mathematical Sciences, Florida Atlantic University, Boca Raton, FL 33431, USA

^+^ Present address: Memorial Sloan Kettering Cancer Center, New York, NY, USA

**Correspondence should be addressed to**: dmitriy.sonkin@nih.gov

**Supplementary table captions**

**Supplemental Table S1.** Exonic fractions for C-terminal and NM_001126112.2 alternative isoforms for each GTEx sample used in analysis.

**Supplemental Table S2.** Exonic fractions for C-terminal and NM_001126112.2 alternative isoforms in TCGA tumors and adjacent normal tissue.

**Supplemental Table S3.** Exonic fractions for C-terminal and NM_001126112.2 alternative isoforms for each TCGA tumor sample used in analysis.

**Supplemental Table S4.** Comparison of exonic fractions for NM_001126112.2 alternative isoform across TCGA tumor types between TP53 WT tumors and TP53 tumors with missense mutations.

**Supplemental Table S5.** Comparison of exonic fractions for NM_001126112.2 alternative isoform across TCGA tumor types between TP53 WT tumors and TP53 tumors with frame shift, nonsense, splice sites mutations.

**Supplemental Table S6.** Exonic fractions for C-terminal and NM_001126112.2 alternative isoforms for each CCLE cell line used in analysis.

**Supplemental Table S7.** Summary of exonic fractions for C-terminal and NM_001126112.2 alternative isoforms in CCLE.
